# Radical-Scavenging Activity and Cytotoxicity of *p*-Methoxyphenol and *p*-Cresol Dimers

**DOI:** 10.3390/molecules15031103

**Published:** 2010-02-26

**Authors:** Yoshinori Kadoma, Yukio Murakami, Takako Ogiwara, Mamoru Machino, Ichiro Yokoe, Seiichiro Fujisawa

**Affiliations:** 1 Institute of Biomaterials and Bioengineering, Tokyo Medical and Dental University, Kanda-surugadai, Chiyoda-ku, Tokyo 101-0062 Japan; E-Mail: y-kadoma.fm@tmd.ac.jp (Y.K.); 2 Meikai University School of Dentistry, Sakado, Saitama 350-0283, Japan; E-Mails: ymura@dent.meikai.ac.jp (Y.M.); a-hirata@dent.meikai.ac.jp (T.O.); machino@dent.meikai.ac.jp (M.M.); 3 Faculty of Pharmaceutical Sciences, Josai University, Saitama 350-0295 Japan; E- Mail: yokoe@josai.ac.jp (I.Y.)

**Keywords:** *p*-cresol dimer, *p*-methoxyphenol dimer, antioxidant, cytotoxicity

## Abstract

Compounds with two phenolic OH groups like curcumin possess efficient antioxidant and anti-inflammatory activity. We synthesized *p-*cresol dimer (2,2'-dihydroxy-5,5'-dimethylbiphenol, **2a**) and *p-*methoxyphenol dimer (2,2'-dihydroxy-5,5'-dimethoxybiphenol, **2b**) by *ortho-ortho* coupling reactions of the parent monomers, *p-*cresol (**1a**) and *p-*methoxyphenol (**1b**), respectively. Their antioxidant activity was determined using the induction period method, and their cytotoxicity towards RAW 264.7 cells was also investigated using a cell counting kit. The stoichiometric factors *n* (number of free radicals trapped by one mole of antioxidant moiety) for **2a** and **2b** were 3 and 2.8, respectively, being greater than those for **1a** and **1b**. The ratio of the rate constant of inhibition to that of propagation (k_inh_/k_p_) for **2a** and **2b** was similar to that for 2-*t*-butyl-4-methoxyphenol (BHA), a conventional food antioxidant. The 50% inhibitory dose (ID_50_) declined in the order **1b** > **1a **>> **2b** > **2a** > BHA. The cytotoxicity for **2a **and **2b** was significantly greater than that for the parent monomers (*p* < 0.001), but smaller than that for BHA (*p* < 0.01). Compounds **2a** and **2b** may be useful as food antioxidants.

## 1. Introduction

**Figure 1:**
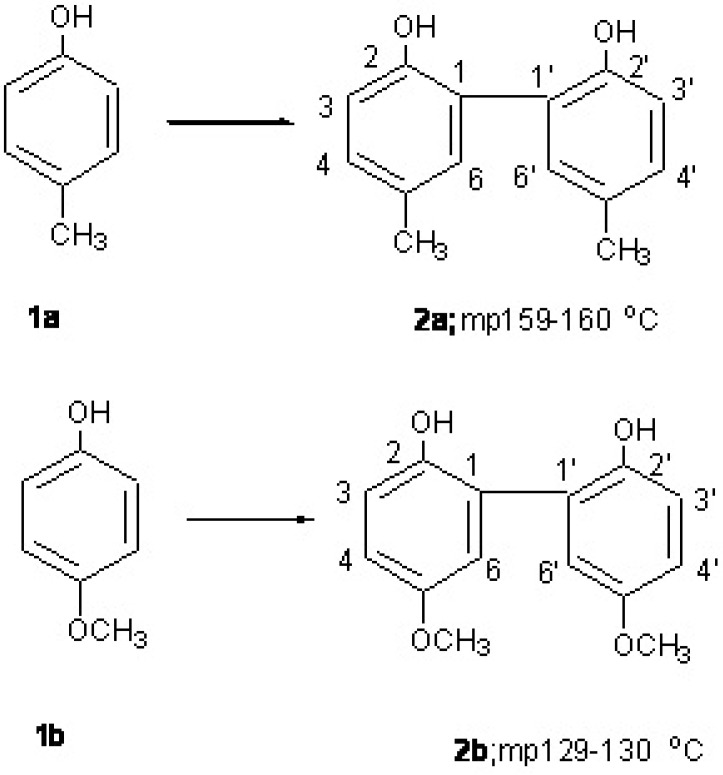
Dimer formation derived from *p*-cresol (**1a**) and *p*-methoxyphenol (**1b**).

*p-*Cresol (**1a**, Figure 1) is a natural product present in many foods, crude oil, and coal tar, and also detectable in animal and human urine. In addition to its industrial uses, **1a** is also used as an antiseptic and disinfectant because of its bactericidal and fungicidal properties, but available data to support the safety of p-cresol for use in cosmetic were insufficient [1]. The oxidative coupling reaction of **1a** with metals in the presence of ethyl acetate, chloroform and hexane produces the dimer, 2,2'-dihydroxy-5,5'-dimethylbiphenol (**2a**) [2]. However, the antioxidant and cytotoxic activities of **2a** remain unclear. *p*-Methoxyphenol (**1b**, Figure 1) is also an acceptable antioxidant, but is known to be carcinogenic, being structurally similar to the known forestomach carcinogen, BHA [3,4]. The adverse toxicological effects of **1b** and BHA may be related to their prooxidative properties. Dimers derived from **1b** and BHA are expected to have enhanced antioxidative activity, because dimerization reduces the prooxidative activity of the parent monomers. 

The coupling reactions of phenol [5], **1b** [6] and BHA [7,8] have been reported previously. We previously synthesized some dimers derived from parent phenolic monomers and investigated their radical-scavenging and biological activities [9,10]. It was found that BHA dimer and phenol dimer (2,2'-biphenol) not only showed enhanced radical-scavenging activity but also anti-inflammatory activity. In the present study, we synthesized the *p-*cresol dimer **2a** (Figure 1) and the *p-*methoxyphenol dimer (2,2'-dihydroxy-5,5'-dimethoxybiphenol, **2b**, Figure 1) derived from the *ortho-ortho* coupling reaction of parent monomers 1a and 1b, respectively. 

The antioxidant activity of phenolic compounds has been assessed using various tests such as inhibition of low-density lipoprotein oxidation [11], the lipid peroxidation capacity (LPIC) assay [12], 1,1’-diphenyl-2-picrylhydrazyl (DPPH)-scavenging assay [13,14], peroxynitrite scavenging assay [13], ABTS (2,2’-azinobis(3-ethylbenzoline-6-sulfonic acid)) radical cation scavenging assay [14,15], DPPH-scavenging assay and the inhibition assay of AAPH (2,2’-azobis(2-amidinopropane) dihydrochloride)-induced peroxidation of linoleic acid in sodium dodecyl sulphate micelles [16] and cupric ion reducing antioxidant capacity(CUPRAC) method [17]. 

In the present study, the radical-scavenging activities were investigated using the induction period method for polymerization of methyl methacrylate (MMA) intiated by thermal decomposition of 2,2’-azobisisobutyronitrile (AIBN) and benzoyl peroxide (BPO). The reaction was monitored by the sensitive method of differential scanning calorimetry (DSC). This induction period method using the AIBN- and BPO-MMA system under air-limited conditions has proved to be reliable for evaluating the antioxidant activity of phenolic compounds [18,19,20]. Also, the ID_50_ for **1a**, **1b**, **2a**, **2b** and BHA towards RAW264.7 cells was investigated. 

## 2. Results and Discussion

### 2.1. Radical-scavenging activity

Typical time-exotherm curves and time-conversion curves for **1a**, **1b**, **2a** and **2b **are shown in Figure 1. The stoichiometric factor (*n*), the ratio of the initial rate of polymerization with an inhibitor to that without an inhibitor (Rp_inh_/Rp_con_), and ratio of the rate constant of inhibition to that of propagation (k_inh_/k_p_) are shown in Table 1. The *n* value for the AIBN system declined in the order **2a** > **2b** > **1b**, BHA > BMP(2-*t*-butyl-4-methylphenol) > BHT(2,6-di-*t*-butyl-4-methylphenol) > **1a**. In contrast, that for the BPO system declined in the order **2a** > **2b** > **1b** > BHA > BHT > BMP > **1a**. The *n* value for **2a** and **2b**, each having two OH groups, was 3 and 2.8 respectively and their value was greater than that for the conventional food antioxidants BHA and BHT. Next we studied the k_inh_/k_p_ values for related phenolic compounds. The k_inh_/k_p_ value for the AIBN system declined in the order **1a** > BHT > BMP > **1b** > **2b** > BHA > **2a**. In contrast, that for the BPO system declined in the order **1a** > BMP > BHT > **1b** > BHA > **2b** > **2a**. In both systems, monophenolic compounds with a 4-methyl group (**1a**, BMP and BHT) showed a larger k_inh_/k_p_ value than those with a 4-methoxy group (**1b** and BHA). This may be related to the finding that the phenolic hydrogen atom in **1b **and BHA is preferentially abstracted, since the *p*-methoxy group is highly activating for abstraction. 1. The k_inh_/k_p_ value for the dimers was smaller than that of the parent monophenols, possibly due to the large stoichoiometric *n* for the dimers.

The k_inh_/k_p_ value for each phenol in the AIBN system was greater than the corresponding one in the BPO system. This may be dependent on the initiation rate (R_i_) of the initiators, because the value for AIBN was about double that for BPO. In the AIBN system, the k_inh_ for **2a**, **2b**, BHA and BHT was 4.7 × 10^3 ^M^-1^s^-1^, 7.1 × 10^3 ^M^-1^s^-1^, 5.8 × 10^3 ^M^-1^s^-1^ and 10.2 × 10^3 ^M^-1^s^-1^, respectively. The k_inh_ for **2a** and **2b** was smaller than that for BHT but the k_inh_ for **2b** was larger than that for BHA (p < 0.01). Note that the k_p_ for MMA of 515 M^-1^s^-1^ at 60 °C was used for that at 70 °C because the k_p_ of MMA at 70 °C is unknown, but was assumed to be close to the value at 60 °C [21]. Generally, the acceptable inhibitors would scavenge a large amount of radicals as well as having a large inhibition rate constant. However, in the present study, phenolic inhibitors such as **2a** and **2b** with a large *n* value showed relatively small inhibition rate constants. 

**Figure 2:**
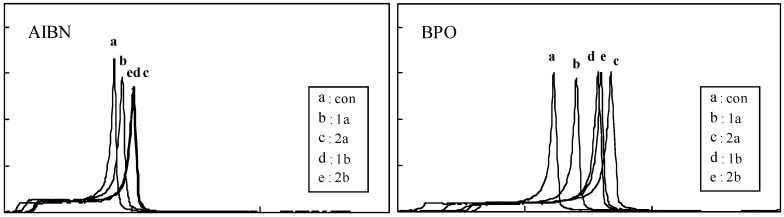
Typical exothermic and time-conversion curves for the polymerization of MMA with AIBN or BPO in the presence of 0.01mol % additives.

**Table 1:** Radical-scavenging activity for *p*-cresol and *p*-methoxyphenol related compounds.

A) AIBN (2,2'-azobisisobutyronitrile)- MMA (methyl methacrylate) system			
**^a^Phenols**	**^b^*n***	**^c^Rp_inh_/Rp_con_**	**^d^k_inh_/k_p_**
*p*-Cresol ( 4-methylphenol, **1a**)	1.6	0.9^*^	18.4
*p*-Cresol dimer (**2a**)	3.0	1.0	9.2
2-*t*-Butyl-4-methylphenol (BMP)	2.3	0.9^*^	14.5
2,6-di-*t*-Butyl-4-methylphenol (BHT)	1.7	1.0	16.5
*p*-Methoxyphenol (**1b**)	2.5	1.0	14.1
*p*-Methoxyphenol dimer (**2b**)	2.8	0.8^**^	11.5
2-*t*-Butyl-4-methoxyphenol (BHA)	2.5	0.9^*^	10.6
**B) BPO (benzoyl peroxide)-MMA system**			
*p*-Cresol ( 4-methylphenol, **1a**)	1.5	1.0	16.6
*p*-Cresol dimer (**2a**)	3.3	1.1	4.2
2-*t*-Butyl-4-methylphenol (BMP)	1.8	1.0	9.3
2,6-di-*t*-Butyl-4-methylphenol (BHT)	1.9	1.0	8.6
*p*-Methoxyphenol (**1b**)	2.4	0.8^**^	8.2
*p*-Methoxyphenol dimer (**2b**)	2.8	0.9^*^	5.7
2-*t*-Butyl-4-methoxyphenol (BHA)	2.2	1.0	6.6

^a ^1 mmol; ^b ^stoichiometric factor; ^c ^the ratio of Rp_inh_ to Rp_con_ ; ^d ^the ratio of k_inh_ to k_p_. Values are the mean of three different experiments. Standard errors < 7%. ^*^p < 0.05 *vs.* control, ^**^ p < 0.01 *vs.* control. MMA, 9.4 mol/L; AIBN (or BPO), 0.1 mol/L; at 70 °C. The procedure is described in the text.

In general, monophenols show an *n* value of 2 [22]. The *n* value for **1a** was 1.5–1.6, suggesting the occurance of dimerization during the induction of polymerization. **1a** may be dimerizable to compounds that can still be effective as antioxidants. When the *n* value is less than 2, particularly around 1, dimerization occurs [22]. The major reaction products from **1a** were previously reported to be the *o-o* dimer, *o-p* dimer and *o-o* trimer, particularly the *o-o* dimer (a 50% yield) [2]. In the present synthetic study, **2a**, the *o-o* dimer, was 40% yield. An oxisodibenzofuran (Pummerer's ketone, *o-p* dimer) derived from *o*-*p* linkage of **1a** was detected but not purified due to the small amount obtained. Also, the *n* value for BHT, particularly in the AIBN system, was less than 2. This suggested dimerization derived from BHT, possibly with formation of stilbenequinone [18,22]. The monophenol inhibitor reacts with two free radicals to give products that are stable with any of the constituents of the reaction mixture. When the reaction gives products that are themselves inhibitors, this could lead to *n* values higher than 2 [22]. In the present study for monophenols, the *n* value of **1b** and BHA for both systems was 2.2–2.5. The *n* value when a methoxy group was present at the *para* position was slightly larger than 2, *i.e.n* for compounds **1b** and BHA, suggesting that this would vary according to the nature of the secondary reactions. The oxidation of 70 °C may proceed rapidly the secondary reaction. On the other hand, when the Rp_inh_/Rp_con _is less than 1_, _the reaction between the carbon-centered radicals of MMA (poly-MMA radicals) and phenoxyl radicals derived from phenolic antioxidants occurs under air-insufficient conditions. In the present study, radical polymerization of MMA was carried out in a DSC pan under air-insufficient conditions, and therefore AIBN and BPO derived alkyl radical (R) and benzyloxy radical (PhCOO), respectively. The decrease in the Rp_inh_/Rp_con _indicated that the growing poly-MMA radicals initiated by AIBN or BPO were suppressed by phenoxyl radicals and/or their intermediates under nearly anaerobic conditions. Note that the oxygen tension under a 15 torr oxygen atmosphere is similar to many tissues, suggesting that oxygen is scare in living cells and that the radical-scavenging activity of antioxidants *in vivo* may differ considerably from that under aerobic conditions.

### 2.2. Cytotoxicity

The cytotoxicity of **1a**, **2a**, **1b** and **2b** towards RAW264.7 cells was estimated within an arbitrary concentration range of 0.00001–10 mM (Figure 2). The ID_50_ (mM) for **1a**, **2a**, **1b** and **2b** was 2.0, 0.6, 2.2, and 0.7, respectively. In contrast, that for BHA was 0.4 mM. The cytotoxicities of **2a** and **2b** were comparable, and significantly higher than those of the parent phenols (p < 0.001). Also, **2a** and **2b **showed slightly smaller cytotoxicity than BHA (p < 0.01).

**Figure 3:**
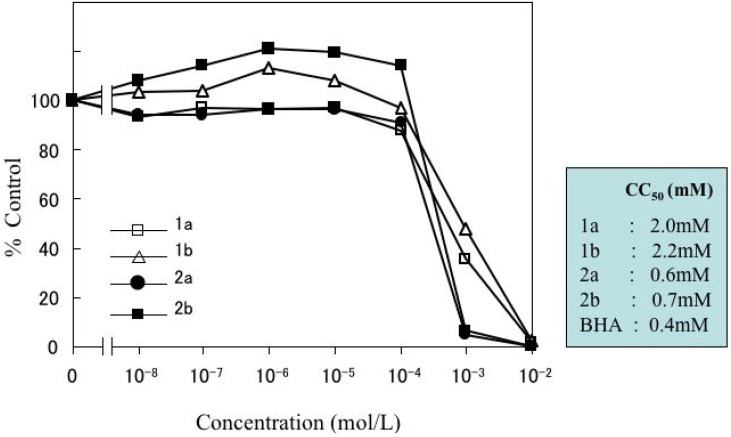
Cytotoxicity of *p*-cresol (**1a**), *p*-cresol dimer (**2a**), *p*-methoxyphenol (**1b**), *p*-methoxyphenol dimer (**2b**) and BHA towards RAW264.7 cells.

We had previously investigated the cytotoxicity of BHA and BHT for human gingival fibroblasts and human HSG cells. For both cell types, the cytotoxicity of BHT was significantly greater than that of BHA [23]. Together from these findings, the cytotoxicity of **2a** and **2b** appeared to be smaller than that of the conventional well-known food antioxidants, BHT and BHA. Compound **1b** is known to be carcinogenic because it is structurally similar to the known forestomach carcinogen BHA [3]. Compound **2b** may suppress the carcinogenic activity because of the alleviation of its prooxidant property caused by dimerization of **1b**. Similarly, BHA reacts with oxidizing agents. It is oxidized by alkaline ferricyanide to 3,3'-di-t-butyl-5,5'-dimethoxy-1,1'-biphenyl-2,2'-diol (bis-BHA) [24]. Also, BHA is a substrate for horseradish and rat intestine mucosa peroxidase by which, in the presence of H_2_O_2_, it is oxidized to bis-BHA [7]. We previously synthesized bis-BHA and reported that this compound was a more effective antioxidant than the parent monomer BHA [20]. Also, we previously reported that bis-BHA shows an anti-inflammatory activity [18]. Recently, we have investigated the inhibitory effect of **1a**, **1b**, **2a** and **2b** on the lipopolysaccharide (LPS)-stimulated cyclooxygenase-2 (COX-2) expression in RAW 264.7 cells. Dimers **2a** and **2b**, but not their monomers, inhibited LPS-stimulated COX-2 expression in RAW 264.7 cells, demonstrating that these compounds possess anti-inflammatory properties (unpublished data). These findings suggest that **2a** and **2b** may be applicable for use as food antioxidants *in vivo*. These results of this study will be reported elsewhere. 

## 3. Experimental

### 3.1. Materials and methods

The following chemicals and reagents were obtained from the indicated companies: **1a**, **1b**, BHA (2-*t*-butyl-4-methoxyphenol), 2-*t-*butyl-4-methylphenol (BMP), 2,6-di-*t*-butyl-4-methylphenol (BHT), MMA (Tokyo Kasei Kogyo, Co., Ltd., Tokyo, Japan). AIBN and BPO (Wako Pure Chemical Industries Ltd. Japan) were recrystallized from chloroform and chloroform/methanol, respectively. NMR spectra were measured in CDCl_3_ solution at ambient temperature on a JEOL, JNM-A500 spectrometer (JEOL, Tokyo, Japan). The chemical shifts δ are given in ppm related to tetramethylsilane, TMS as internal standard. Mass spectra (MS) were measured on a JMS/700 (JEOL).

### 3.2. Synthesis of phenol dimers

*Synthesis of 2,2'-dihydroxy-5,5'-dimethylbiphenol* (**2a**): A solution of the 4-methylphenol (**1a**,1.08 g, 0.01 mol) in nitromethane (20 mL) was added to a solution of AlCl_3 _(1.33 g, 0.01 mol) in nitromethane (20 mL) under nitrogen. After 30 min, a solution of anhydrous FeCl_3_ (1.62 g, 0.01 mol) in nitromethane (20 mL) was added, and mixing was continued for 5 h at room temperature. The reaction was quenched with 10% HCl and the reaction mixture was extracted with CH_2_Cl_2_.The organic phase was dried (Na_2_SO_4_), the solvent was evaporated, and the residue was chromatographed on silica gel with a *n*-hexane-EtOAc mixture to give dimer **2a**, mp 159-160 °C, 40% yield. MS m/z; 214 (M^+^), ^1^H-NMR（CDCl_3_, 500MHz; δ(ppm) 2.32 (s, 6H, 2CH_3_), 5.34 (br s, 2H, 2OH) 6.92 (d, 2H, H-3 and H-3’, *J* = 8.2 Hz), 7.06 (dd, 2H, H-6 and H-6’, *J* = 2.2), 7.11(dd, 2H, H-4 and H-4’, *J* = 8.2 and 2.2 Hz).

*Synthesis of 2,2'-dihydroxy-5,5'-dimethoxybiphenol* (**2b**): In accordance with the above procedure, 4-methoxyphenol dimer was obtained (80% yield). Mp 129–130 °C, MS m/z; 246 (M^+^), ^1^H-NMR (CDCl_3_, 500MHz; δ(ppm) 3.80 (s, 6H, 2OCH_3 _), 5.02 (br s, 2H, 2OH), 6.89 (d, 2H, H-3 and H-3’, *J =* 8.8 Hz), 6.63 (d, 2H, H-6 and H-6’, *J =* 2.7 Hz), 6.71 (dd, 2H, H-4 and H-4’, *J =* 8.8 and 2.7 Hz).

### 3.3. Induction time (IT) and initial rate of polymerization (Rp)

The induction time and initial rate of polymerization in the presence (Rp_inh_) or absence (Rp_con_) of an antioxidant were determined by the method reported previously [18,19,20]. In brief, the experimental resin consisted of MMA and AIBN or BPO with or without additives. AIBN or BPO was added at 1.0 mol %, and the additives were used at 0.01 mol %. Approximately 10 μL of the experimental resin (MMA: 9.15–9.30 mg) was loaded into an aluminum sample container and sealed by applying pressure. The container was placed in a DSC (model DSC 3100; Mac Science Co., Tokyo, Japan) kept at 70 °C, and the thermal changes induced by polymerization were recorded for the appropriate periods. The heat due to the polymerization of MMA was 13.0 kcal mol^-1^. The conversion of all samples (%) was calculated from the DSC thermograms using the integrated heat evoked by polymerization of MMA. The value was 92.8–95.9%. Time-exotherm and time-conversion curves of **1a, 1b, 2a** and **2b** are shown in Figure 1. Time-conversion curves break when a phenolic inhibitor is consumed. These breaks are sharp and provide a reliable measure of the induction time of the inhibitor. The induction time (IT) was calculated from the difference between the IT of inhibitors and that of controls. The initial rates of polymerization in the absence (Rp_con_) and presence (Rp_inh_) of a phenolic inhibitor were calculated from the slope of the plots during the initial linear phase of the conversion rate of MMA polymerization (tangent drawn at the early polymerization stage), as reported previously [18,19].

### 3.4. Measurement of stoichiometric factor (n)

The *n* value in Equation (1) can be calculated from the induction time in the presence of inhibitors:

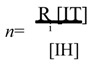
(1)
where IT is the induction time in the presence of an inhibitor (IH). The number of moles of peroxy radicals trapped by the antioxidant was calculated with respect to 1 mole of inhibitor moiety unit. The R_i_ values for AIBN and BPO at 70 °C were 6.40 × 10^-6 ^and 2.28× 10^-6 ^mol l^-1^s^-1^, respectively.

### 3.5. Measurement of the inhibition rate constant (k*inh*)

When R_i_ is constant, *i.e.* when new chains are started at a constant rate, a steady-state treatment can be applied and the initial rate of polymerization of MMA is given by Equation (2):

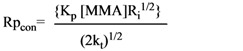
(2)
where MMA represents methyl methacrylate and k_p_ and k_t_ are the rate constants for chain propagation and termination, respectively. The Rp_inh_ rates are determined by Equation (3):

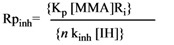
(3)
in which Rp_inh_ is the initial rate of inhibited polymerization, [MMA], *n*, [IH] and k_p_ are defined above, and k_inh_ is the rate constant for scavenging (inhibition) of MMA radicals by an antioxidant. From Equation (2) and Equation (3), k_inh_/k_p_ can be calculated by Equation (4):

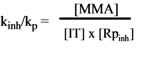
(4)


### 3.6. Cytotoxicity

The murine macrophage-like cell line RAW 264.7 is established from the ascites of a tumour induced in a male mouse by intraperitoneal injection of Abselon Leukaemia Virus (A-MuLV). We used RAW 264.7 cells (Dainippon Sumitomo Pharma Biomedical Co. Ltd., Osaka, Japan) for the cytotoxicity test. The cells were cultured to a subconfluent state in RPMI-1640 (Sigma-Aldrich Co., Japan) medium supplemented with 10% FBS at 37 °C and 5% CO_2_ in air, washed and then incubated overnight in serum-free RPMI-1640. They were then washed further and treated with the test samples.

The relative number of viable cells was determined by a Cell Counting Kit-8 (CCK-8) (Dojindo Co., Kumamoto, Japan) (10). In brief, RAW 264.7 cells (3 × 10^4^ per well) were cultured in NUNC 96-well plates (flat-well-type microculture plates) for 48 hours, after which the cells were incubated with test samples for 24 hours. CCK-8 solution was added to each well and then the absorbance was measured at 450 nm with a microplate reader (Biochromatic, Helsinki, Finland). The ID_50_ was determined from the dose-response curves. Data were expressed as means of three independent experiments. Standard errors <15%. Statistical analyses were performed by Student’s *t*-test.

## 4. Conclusions

We synthesized the related compounds **2a** and **2b** and investigated their radical-scavenging activity using the induction period method. Their *n* values were 3 and 2.8, respectively, the largest values among related compounds. The k_inh_/k_p_ values for **2a** and **2b** were similar to that of BHA, a conventional food antioxidant. The cytotoxicity of dimers **2a** and **2b** was less than that of BHA. These dimers may be applicable as food antioxidants. However, further safety testing would be needed before use in a food antioxidant application.

## References

[1] Andersen A. (2006). Final report on the safety assessment of sodium *p*-chloro-*m*-cresol, *p*-chloro-*m*- cresol, chlorothymol, mixed cresols, *m*-cresol, *o*-cresol, *p*-cresol, isopropyl cresols, thymol, *o*- cymen-5-ol, and carvacrol. Int. J. Toxicol..

[2] Asakura K., Honda E., Osanai S. (1995). Selective oxidative coupling of *p*-cresol producing an *ortho-ortho* direct-linked dimer. Chem. Lett..

[3] Asakawa E., Hirose M., Hagiwara A., Takahashi S., Ito N. (1994). Carcinogenicity of 4-methoxyphenol and 4-methylcatechol in F344 rats. Int. J. Cancer..

[4] Shibata M., Hirose M., Kagawa M., Boonyaphiphat P., Ito N. (1993). Enhancing effect of concomitant *L*-ascorbic acid administration on BHA-induced forestomach carcinogenesis in rats. Carcinogenesis.

[5] Danner D.J., Brignac P.J., Arceneaux D., Patel V. (1973). The oxidation of phenol and its reaction product by horseradish peroxidase and hydrogen peroxide. Arch. Biochem. Biophys..

[6] Sartori G., Maggi R., Bigi F., Arienti A., Casnati G., Bocelli G., Mori G. (1992). Oxidative coupling of dichloroaluminium phenolates: Highly selective synthesis of hydroxylated bi- and tetraaryls. Tetrahedron.

[7] Sgaragli G., Corte L.D., Puliti R., De Sarlo F., Francalanci R., Guarna A. (1980). Oxidation of 2-*t*-butyl-4-methoxyphenol (BHA) by horseradish and mammalian peroxidase systems. Biochem. Pharmacol..

[8] Fujisawa S., Atsumi T., Kadoma Y., Sakagami H. (2002). Antioxidant and prooxidant action eugenol-related compounds and its cytotoxicity. Toxicology.

[9] Murakami Y., Shoji M., Hirata A., Tanaka S., Hanazawa S., Yokoe I., Fujisawa S. (2006). An *ortho* dimer of butylated hydroxyanisole inhibits nuclear factor kappa B activation and gene expression of inflammatory cytokines in macrophages stimulated by *Porphyromonas gingivalis* fimbriae. Arch. Biochem. Biophys..

[10] Murakami Y., Ishii H., Hoshina S., Takada N., Ueki A., Tanaka S., Kadoma Y., Ito S., Machino M., Fujisawa S. (2009). Antioxidant and cyclooxygenase-2-inhibiting activity of 4,4’-biphenol, 2,2’-biphenol and phenol. Anticancer Res..

[11] Leenen R., Roodenburg A.J., Vissers M.N., Schuurbiers J.A., van Putte K.P., Wiseman S.A., van de Put F.H. (2002). Supplementation of plasma with olive oil phenols and extracts: influence on LDL oxidation. J. Agric. Food Chem..

[12] Zhang J., Stanley R.A., Melton L.D., Skinner M.A. (2007). Inhibition of lipid oxidation by phenolic antioxidants in relation to their physicochemical properties. Pharmacologyonline.

[13] Lacikova L., Jancova M., Muselik J., Masterova I., Grancai D., Fickova M. (2009). Antiproliferative, cytotoxic, antioxidant activity and polyphenols contents in leaves of four *Staphylea L.* specie. Molecules.

[14] Modak B., Rojas M., Torres R., Rodilla J., Luebert F. (2007). Antioxidant activity of a new aromatic geranyl derivative of the resinous exudates from Heliotropium glutinosum Phil. Molecules.

[15] Ertan Anli R., Vural N. (2009). Antioxidant phenolic substances of Turkish red wines from different wine regions. Molecules.

[16] Roche M., Dufour C., Mora N., Dangles O. (2005). Antioxidant activity of olive phenols: mechanistic investigation and characterization of oxidation products by mass spectrometry. Org. Biomol. Chem..

[17] Apak R., Güçlü K., Demirata B., Ozyürek M., Celik S.E., Bektaşoğlu B., Berker K.I., Ozyurt D. (2007). Comparative evaluation of various total antioxidant capacity assays applied to phenolic compounds with the CUPRAC assay. Molecules.

[18] Fujisawa S., Kadoma Y., Yokoe I. (2004). Radical-scavenging activity of butylated hydroxytoluene (BHT) and its metabolites. Chem. Phys. Lipids.

[19] Kadoma Y., Atsumi T., Okada M., Ishihara M., Yokoe I., Fujisawa S. (2007). Radical-scavenging activity of natural methoxyphenols *vs.* synthetic ones using the induction period. Molecules.

[20] Kadoma Y., Ito S., Yokoe I., Fujisawa S. (2008). Comparative study of the alkyl and peroxy radical-scavenging activity of 2-*t*-butyl-4-methoxyphenol (BHA) and its dimer and their theoretical parameters. In Vivo.

[21] Odian G.G. (2004). Principles of Polymerization.

[22] Horswill E.C., Howards J.A., Ingold K.U. (1966). he oxidation of phenols. III. The stoichiometries for the oxidation of some substituted phenols with peroxy radicals. Can. J. Chem..

[23] Kadoma Y., Ito S., Atsumi T., Fujisawa S. (2009). Mechanisms of cytotoxicity of 2- or 2,6-di-*tert*-butylphenols and 2-methoxyphenols in terms of inhibition rate constant and a theoretical parameter. Chemosphere.

[24] Hewgill F.R., Hewitt D.G. (1967). Oxidation of alkoxyphenol. Part X. The reaction of 2,2’-dihydroxy-5,5’-dimethoxy-3, 3’-di-*t*-butylbiphenyl with lead tetra acetate. J. Chem. Soc. C.

